# Phytochemical profiling and anticancer activity of the n-butanol fraction from Ardisia villosa extract: Inhibition of gastric cancer cell proliferation via cell cycle arrest and senescence induction

**DOI:** 10.1371/journal.pone.0340458

**Published:** 2026-01-08

**Authors:** Nguyet Mai Hua, Van Khang Pham, Thi Thanh Huong Le, Son Hiep Pham, Viet Hoang, Thu Huong Trinh, Dinh Quang Hung Can, Van Hung Hoang, Phu Hung Nguyen

**Affiliations:** 1 Faculty of Natural Science and technology, TNU- University of Sciences, Thai Nguyen Province, Vietnam; 2 Faculty of Chemistry, TNU - University of Education, Thai Nguyen Province, Vietnam; 3 Institute for Excellence in Education and Research, Thai Nguyen University (TNU), Thai Nguyen Province, Vietnam; 4 Thai Nguyen University, Thai Nguyen province, Vietnam; University of Bergen: Universitetet i Bergen, NORWAY

## Abstract

Medicinal plants serve as valuable sources for anticancer drug discovery. This study investigated the anticancer potential of the *n*-butanol fraction from Ardisia villosa extract against breast and gastric cancer cell lines. Phytochemical profiling using UPLC-QToF-MS in both positive and negative ESI modes identified 118 putative compounds, including flavonoids, lignans, alkaloids, triterpenoids, steroids, coumarins, and phenolic acids. The *n*-butanol fraction exhibited dose-dependent antiproliferative effects, with IC_50_ values of 60.2 µg/mL (MCF-7), 85.2 µg/mL (MKN45), and 51.7 µg/mL (AGS). In AGS gastric cancer cells, the extract significantly inhibited 3D tumorsphere formation and suppressed cell migration at concentrations as low as 50 µg/mL (p < 0.001). Additionally, *n*-butanol fraction extract markedly induced cellular senescence (p < 0.01). Mechanistic investigations revealed that the extract induced G_0_/G1 phase arrest by downregulating critical cell cycle regulators, including CCND1, CCNE1, CDK2, CDK3, CDK6, CDK8, and CDK9, while upregulating tumor suppressor and senescence-related genes such as p21, p53, p16, and p27 (p < 0.01). Molecular docking analyses further supported these findings by demonstrating strong binding affinities of phytochemicals to key cell cycle regulatory proteins, suggesting a direct molecular basis for their antiproliferative effects. In conclusion, the *n*-butanol fraction of *Ardisia villosa* displays potent anticancer activity, particularly in gastric cancer cells, through multi-targeted mechanisms involving cell cycle inhibition and senescence induction, and holds promise as a natural source for future anticancer therapeutics.

## Introduction

Globally, gastric cancer remains one of the most prevalent malignancies and has consistently ranked as the fourth leading cause of cancer-related mortality over the past several years [1]. The incidence and mortality rates associated with gastric cancer are steadily increasing, with a notable trend toward younger patient populations [2,3]. Prominent risk factors include a family history of gastric cancer, dietary habits, tobacco smoking, and infections with *Helicobacter pylori* and Epstein–Barr virus (EBV [4].

Surgical resection, chemotherapy, and radiotherapy continue to be the most commonly employed treatment modalities in most countries. Among these methods, surgical intervention seeks to completely remove the tumor; however, it involves inherent risks, such as the possibility of tumor cells entering the bloodstream, which could eventually result in metastasis [5]. Moreover, surgery often involves a prolonged recovery period and is not feasible for patients with advanced metastatic disease. Chemotherapy serves as a primary therapeutic strategy, especially in poorly differentiated gastric cancer, effectively inhibiting tumor progression and reducing tumor mass prior to surgical intervention. Nevertheless, it frequently exerts deleterious effects on patient health due to its severe side effects [6].

The toxic side effects associated with conventional therapies have stimulated research into the development of novel therapeutic agents that offer high efficacy with lower toxicity [7]. In this context, the exploration of natural compounds derived from medicinal plants, with inherent biocompatibility, anticancer potential, and minimal side effects has emerged as a promising research direction [8].

The genus *Ardisia*, comprising approximately 500 species predominantly distributed in tropical and subtropical regions, is widely utilized as ornamental plants, food sources, and traditional medicines [9]. Various studies have demonstrated that species within this genus contain a diverse range of bioactive compounds, including polyphenols, triterpenoid saponins, quinones, and alkylphenols [10]. Several *Ardisia* species have exhibited cytotoxic activities against different cancer cell lines, such as hepatocellular carcinoma, breast cancer, gastric cancer, lung cancer, and colorectal cancer [11,12]. Consequently, *Ardisia* is considered a valuable resource for the development of novel anticancer therapeutics.

*Ardisia villosa*, a species within the *Ardisia* genus, is found across various regions, including mountainous areas in Northern Vietnam. Phytochemical analyses have revealed the presence of compounds such as triterpenoid saponins, quinones, phenolics, coumarins, cyclic depsipeptides, and flavonoids in *Ardisia* species, which have been associated with anticancer, anti-inflammatory, and antimicrobial properties [13]. Previous studies have also indicated that *Ardisia villosa* possesses inhibitory effects on the proliferation of gastric cancer cells [14]. In traditional medicine, several species of the genus *Ardisia*, including *Ardisia villosa*, have been used as important components in herbal formulations for the treatment of rheumatoid arthritis and for relieving bruises and injuries. In addition, it has also been reported to be used in traditional remedies for the treatment of diseases such as dysentery, pulmonary tuberculosis, haemoptysis, and haematemesis [10].

Although preliminary assessments have suggested the cytotoxic potential of *Ardisia villosa*, the anticancer activity of its extracts and the underlying mechanisms of action remain largely unexplored. Therefore, the objective of the present study is to investigate the anticancer effects of the *n*-butanol fraction of *Ardisia villosa* on AGS human gastric cancer cells.

## Materials and methods

### Preparation of the *n*-butanol fraction from ardisia villosa leaf extract

**Fig 1:**
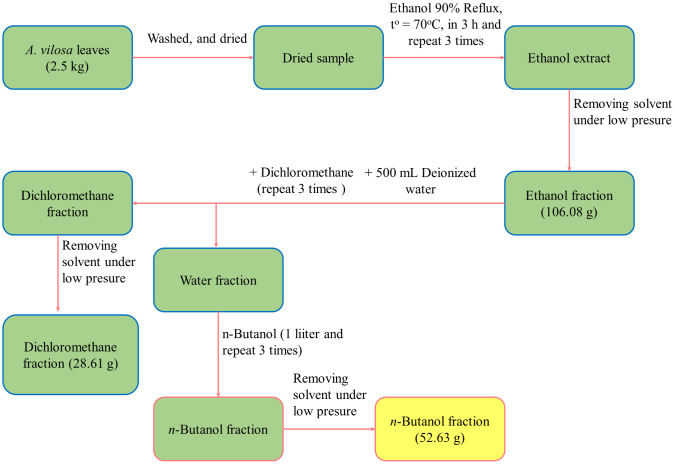
Fractionation scheme of the butanol extract from *Ardisia gigantifolia* leaves.

*Ardisia villosa* leaves (voucher TNU2021.07) were gathered in Thai Nguyen Province, Vietnam, and was taxonomically identified by plant taxonomy expert Dr. Thi Thanh Huong Le (Thai Nguyen University) based on the morphological method. Then, the fresh leaves were accurately weighed to 2.5 kg before washing and air-drying. Subsequently, the dried sample was sequentially extracted with 95% ethanol, dichloromethane, deionized water and *n*-butanol, with each extraction cycle performed in triplicate under identical conditions for each solvent. Notably, ethanol extraction was conducted at 70 °C for three hours each cycle. Finally, filtrates were combined and concentrated under reduced pressure in a rotary evaporator to ensure complete removal of all solvents (Fig 1).

### Phytochemical profiling of *n*-butanol fraction

The metabolite profiling of the butanol extract and its subfractions of *Ardisia villosa* was performed using an ACQUITY UPLC I-Class Plus system coupled with a Xevo G3 ESI/QTOF high-resolution mass spectrometer (Waters Corporation, USA) [15]. Samples were prepared by dissolving 5 mg of dried extract in 0.5 mL of methanol, filtered through a 0.22 µm membrane, and 1 µL was injected for chromatographic separation using a BEH C18 column. Mass detection was conducted in both positive and negative electrospray ionization modes under optimized conditions. Data acquisition was carried out in full scan mode (m/z 50–1100), and fragmentation information was obtained using low- and high-energy modes. Spectral data were processed using UNIFI software, and peak annotation was performed based on fragmentation patterns compared against the Waters Traditional Medicine Library (Waters Corporation, USA).

### Cell culture and MTT assay

MCF7 (breast cancer cells), along with MKN45 and AGS (gastric cancer cells), were seeded into 96-well plates at a density of 1 × 10^4^ cells per well in 100 µL of RPMI 1640 medium, which was enhanced with 10% FBS and 1% ampicillin/streptomycin. The cells were then incubated at 37 °C in a humidified environment containing 5% CO2. After allowing 16–24 hours for cell adhesion, cells were treated with varying concentrations of the butanol extract or its subfractions, specifically 0, 10, 50, 100, 200, or 500 µg/mL. After 48 hours of exposure, morphological alterations were observed and recorded using phase contrast microscopy with a Nikon Ts2 inverted microscope.

The MTT assay was conducted following the reviously protocol [16]. After discarding the spent medium, each well received 100 µL of fresh medium containing MTT at 0.5 mg/mL (Thermo Fisher Scientific, US), and were incubated at 37 °C in 5% CO₂ for 4 h. Thereafter, the MTT solution was removed and replaced with 100 µL of dimethyl sulfoxide (VWR, France), followed by a 15 minutes incubation at 37 °C to solubilize the formazan crystals. Cell viability was assessed by measuring absorbance at 570 nm on a Multiskan Sky spectrophotometer (Thermo Fisher Scientific, USA). Proliferation was expressed as a percentage using the formula provided above.


$$\%\;\text{Cell}\;\text{proliferation}=\frac{\text{OD}\;\text{of}\;\text{the}\;\text{treated}\;\text{sample}}{\text{OD}\;\text{of}\;\text{the}\;\text{control}}\;\times \text{1}\text{0}\text{0}$$

All assays were conducted in triplicate across three independent experiments. IC_50_ values for cell proliferation were determined by nonlinear regression using GraphPad Prism 10.5 in accordance with the manufacturer’s guidelines.

### Cell migration analysis

Cells were plated in 96-well plates (100 µL/well; 2.0 × 10⁵ cells/well) and allowed to form a uniform monolayer. A linear scratch was made using a 10-µL pipette tip to generate a cell-free gap. Debris and detached cells were removed by washing twice with PBS. The cultures were then exposed to n-buthanol fraction extract (50 and 100) for 24 h. Gap closure, used as an index of migratory capacity, was imaged with a Nikon Ts2 inverted microscope (Tokyo, Japan) and quantified in ImageJ by measuring the gap width at each time point [17]. The remaining open area was expressed as:


$$\text{Open}\;\text{area}\;(\%)=\frac{\text{Width}\;\text{at}\;\text{24}\;\text{h}}{\text{Width}\;\text{at}\;\text{0}\;\text{h}}\;\times \text{1}\text{0}\text{0}$$

### Tumorsphere formation assay

To generate non-adherent culture conditions, 24- or 96-well plates were coated with poly(2-hydroxyethyl methacrylate) (polyHEMA; 10 mg/mL in 96% ethanol). Briefly, 50 µL (96-well) or 100 µL (24-well) polyHEMA solution was dispensed per well, dried by complete ethanol evaporation at 50–60°C, and the coating step was repeated twice. Wells were then rinsed three times with PBS. During washing and medium changes, pipetting was performed gently to avoid disrupting the polyHEMA layer.

Cells (1,000 cells/well) were seeded in DMEM/F-12 supplemented with 1% penicillin/streptomycin, 0.1% amphotericin B (500 µg/mL), 20 ng/mL EGF, and 0.4% glucose, and treated with ACE at indicated concentrations. After 48 h, tumorspheres were imaged using a Nikon Eclipse Ti2 inverted microscope. Sphere number (clusters ≥ 10 cells) was counted at 100 × –200 × , and sphere diameter was measured from 200 × images using ImageJ [17]. Data were expressed as percentages relative to the vehicle control.

### Cell cycle analysis

Cell cycle distribution was assessed utilizing a BD Accuri™ C6 Plus flow cytometer (BD Biosciences, USA) as previously described [18]. A total of 2 × 10^^4^ cells were seeded in 24-well plates and incubated in RPMI 1640 medium for 24 hours. Subsequently, the cells were treated with fresh medium containing subfraction of butanol extract at varying concentrations ranging from 0 to 200 µg/mL for a duration of 48 hours. Post-treatment, the cells were fixed in 70% ethanol and stained with propidium iodide (PI) for 30 minutes. Following staining, cells were collected through trypsinization and centrifuged at 13,000 rpm for 5 minutes. Flow cytometric analysis was conducted to evaluate DNA content and cell cycle phases, with all experiments performed in triplicate to ensure reproducibility [18].

### Cell senescence assay

Senescence was evaluated through histochemical staining using Senescence Cells Histochemical Staining Kit (Sigma-Aldrich, France), according to the manufacturer’s protocol. The cells were initially washed twice with 1 × phosphate buffered saline (PBS) solution to remove any residual medium or debris. Subsequently, cells were fixed with 1 × Fixation buffer and incubated the plate for 10–15 minutes at room temperature to preserve their structural integrity. After fixation, the Fixation buffer was discarded, and the cells were rinsed twice more with 1 × PBS, using 500 µL per wash to ensure complete removal of the fixative. Subsequently, 50 µL Staining mix solution containing senescence-associated β-galactosidase (SA-β-gal) was applied to each well. The cells were then cultured at 37°C in the absence of light for 2 hours to facilitate the detection of SA-β-gal activity, which serves as a marker for cellular senescence. Following the incubation period, the cells were examined under a light inverted microscope at 100 × magnification. The presence of senescent cells was confirmed by the characteristic blue staining, which was visualized and documented through microscopic imaging [19]. The experiments were repeated 3 times for each condition

### Real-time PCR analysis for mRNA expression

Cancer cells were cultured in 6-well plates at a density of 2 × 10⁵ cells per well and subjected to a subfraction of butanol extract at its IC_50_ concentration for a duration of 24 h. Subsequent to the treatment, total RNA was extracted utilizing TRIzol reagent (Invitrogen Thermo Fisher Scientific, US) in accordance with established protocols. Following chloroform-induced phase separation and ethanol-mediated RNA precipitation, the RNA was washed with 70% ethanol, air-dried, and reconstituted in nuclease-free water. The yield and purity of RNA were quantified using a NanoDrop spectrophotometer. Quantitative real-time PCR were conducted utilizing the BIOFACT 2 × One-Step SYBR Green RT-qPCR kit (BIOFACT, Korea) on a qTower³ thermal cycler (Analytik Jena, Germany), with each reaction comprising 20 ng of total RNA. The thermal profile included an initial denaturation at 95 °C for 15 minutes, followed by 95 °C for 20 seconds and 60 °C for 20 seconds for 40 cell cycles. Primer sequences used are listed in S1 Table. HPRT1 was used as the reference gene, and relative expression was calculated using the 2^⁻ΔΔCt^ method [20]. All experiments were conducted in three independent replicates.

### Molecular docking

#### Ligand and protein preparation.

The chemical structures of the investigated compounds were either retrieved from the PubChem database (https://pubchem.ncbi.nlm.nih.gov) in SDF format or manually drawn using ChemDraw 23.1.1. Ligands were prepared using the LigPrep module in Schrödinger Maestro (Release 2024−3), which generated all plausible tautomeric and stereoisomeric forms, assigned ionization states at pH 7.0 ± 2.0, and optimized each structure to its lowest-energy 3D conformation using the OPLS4 force field.

The crystal structures of cyclin-dependent kinases CDK2, CDK3, CDK4, CDK6, CDK8, and CDK9 were retrieved from the Protein Data Bank (PDB IDs: 2R3I, 8H4R, 7SJ3, 5L2S, 5HBJ, and 8I0L, respectively). Protein structures were processed using the Protein Preparation Wizard. During preparation, bond orders were reassigned, missing hydrogens were added, and crystallographic water molecules not involved in ligand interactions were removed. The protonation states of ionizable residues were predicted using PROPKA at pH 7.4, and hydrogen-bonding networks were optimized accordingly. For co-crystallized ligands and hetero groups, Epik was used to determine protonation and tautomeric states at pH 7.4 ± 2.0.

#### Molecular docking and validation.

For each CDK target, the binding site was defined based on the location of the co-crystallized ligand in the corresponding PDB structure. The receptor grid boxes used for docking were centered on this ligand and adjusted to encompass the entire binding pocket. Grid coordinates and dimensions for each protein target are listed in Table 1.

**Table 1:** Receptor grid box parameters for CDK proteins.

Grid box parameters	CDK2	CDK3	CDK4	CDK6	CDK8	CDK9
**Center_x**	1.932	24.472	11.657	21.986	−4.633	53.649
**Center_y**	27.635	−98.257	−39.053	38.630	12.823	−17.358
**Center_z**	7.948	12.691	10.364	−9.236	−11.182	−12.352
**Innerbox_x**	10	10	10	10	10	10
**Innerbox_y**	10	10	10	10	10	10
**Innerbox_z**	10	10	10	10	10	10
**Outerbox_x**	23.434	23.969	26.622	26.521	23.957	24.671
**Outerbox_y**	23.434	23.969	26.622	26.521	23.957	24.671
**Outerbox_z**	23.434	23.969	26.622	26.521	23.957	24.671

To validate the docking protocol, the co-crystallized ligands were re-docked into their original binding sites using the Glide XP (extra precision). The resulting poses were compared with the crystal structures by calculating the root-mean-square deviation (RMSD), and docking was considered reliable when RMSD values were below 2.0 Å. Subsequently, 118 prepared ligands were docked against all six CDK targets using Glide XP.

#### MM-GBSA binding free energy estimation.

The binding free energies of the top-ranked docking poses were evaluated using the Prime MM-GBSA method (Molecular Mechanics with Generalized Born Surface Area), which is widely recognized as a robust method for estimating relative binding affinities of ligand–protein complexes [21]. Calculations were performed using the VSGB solvation model and the OPLS4 force field. Binding free energy (ΔG_bind_) was calculated as:


$$\Delta {\text{G}}_{\text{bind}}={\text{G}}_{\text{complex}}-{\text{G}}_{\text{protein}}-{\text{G}}_{\text{ligand}}$$

where *G*_*complex*_ represents the free energy of the complex, and *G*_*protein*_ and *G*_*ligand*_ represent the MM-GBSA energies of the unbound protein and ligand, respectively.

### Data analysis

The data gathered were examined using the specialized software GraphPad Prism 10.5 (GraphPad Software, San Diego, CA, USA). Mann-Whitney and one-way ANOVA test were employed for statistical analysis, with a p-value of less than 0.05 considered as statistically significant.

## Results

### Phytochemical profiling of the *n*-butanol fraction

**Fig 2:**
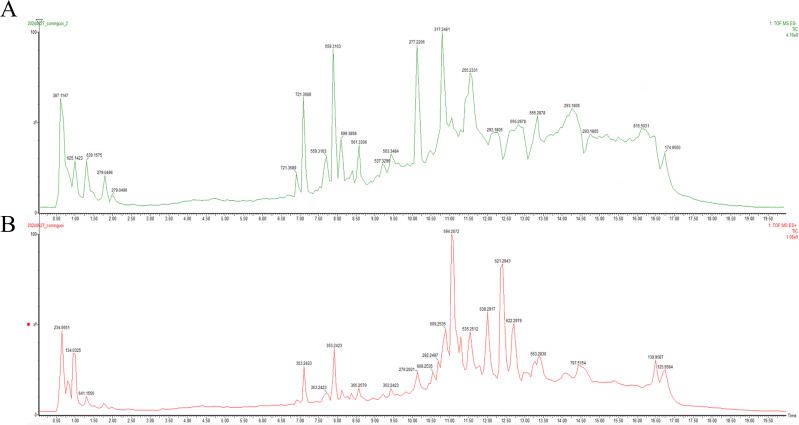
UPLC-QToF-MS chromatographic profiles of *n*-butanol soluble fractions isolated from *Ardisia villosa.* Extract under (A) negative and (B) positive ESI modes.

Compound identification was performed using exact mass measurements, fragmentation patterns obtained under both high- and low-energy collision conditions, and spectral matching with library data. The respective total ion chromatograms (TICs) are shown in Fig 2 which illustrates the chromatograms produced under two ionization conditions: (A) negative and (B) positive. Positive electrospray ionization (ESI) mode exhibited superior ionization efficiency, as indicated by a greater number of detected ion signals compared to the negative ion mode. During data preprocessing, only peaks with signal intensities above 5000 counts were annotated, and a mass error tolerance of 5 ppm was applied to match experimental data with theoretical values.

The compounds were arranged according to observed RT. A total of one hundred and eighteen compounds were tentatively identified including ten flavonoid compounds, ten lignans, twenty-two alkaloids, thirty-seven triterpenoids, thirteen steroids, 5 coumarin compounds, twelve phenolic & aromatic compounds and other funtional ingredients. It is possible to check the distribution of the determined secondary metabolites in the analyzed sample. The 118 detected compounds with UPLC-QToF-MS in both negative and positive mode are listed in Table 2, along with names of the compounds; formula; expected mass (Da); observed mass (Da); observed (m/z); mass error (mDa); observed RT (min); class; pathway.

**Table 2:** Phytochemical compound of *n*-butanol fraction from *Ardisia villosa.*

No.	Compound	Formula	Expected mass (Da)	Observed mass (Da)	Observed (m/z)	Mass Error (mDa)	Observed RT (min)	Class	Pathway
1	**Oleracein D**	C_31_H_37_NO_17_	695.206	695.205	696.213	−0.782	0.665	Betalain alkaloids	Alkaloids
2	**Apiosylskimmin**	C_20_H_24_O_12_	456.127	456.126	457.134	−0.498	0.665	Simple coumarins	Shikimates and Phenyl-propanoids
3	**Ononin**	C_22_H_22_O_9_	430.126	430.126	431.133	−0.655	0.665	Isoflavones	Shikimates and Phenyl-propanoids
4	**Decursitin F**	C_16_H_16_O_6_	304.095	304.096	305.103	1.412	0.666	Pyrano-coumarins	Shikimates and Phenyl-propanoids
5	**Nordracorubin**	C_31_H_22_O_5_	474.147	474.146	475.154	−0.362	0.671	Flavans	Shikimates and Phenyl-propanoids
6	**Bruceene**	C_20_H_26_O_8_	394.163	394.162	395.170	−0.417	0.674	Quassinoids	Terpenoids
7	**Podophyllotoxin**	C_22_H_22_O_8_	414.131	414.133	415.140	1.534	0.675	Aryl-naphthalene	Shikimates and Phenyl-propanoids
8	**1,7-Bis(4-hydroxyphenyl)-hepta-4E,6E-dien-3-one**	C_19_H_18_O_3_	294.126	294.127	295.134	1.211	0.729	Linear diaryl-heptanoids	Shikimates and Phenyl-propanoids
9	**n-(1,7-dimethoxy-phenanthren-2-yl)-acetamide**	C_18_H_17_NO_3_	295.121	295.122	296.129	0.914	0.730	Phenanth-renes	Shikimates and Phenyl-propanoids
10	**Xantholide A**	C_15_H_18_O_2_	230.131	230.130	231.138	−0.379	0.846	Guaiane sesqui-terpenoids	Terpenoids
11	**Kushenol W**	C_21_H_22_O_7_	386.137	386.137	387.145	0.706	0.868	Flavanones	Shikimates and Phenyl-propanoids
12	**Leonticine**	C_20_H_25_NO_3_	327.183	327.182	328.190	−1.011	0.868	Monomeric stilbenes	Shikimates and Phenyl-propanoids
13	**Didemethoxyl-curcumin**	C_19_H_16_O_4_	308.105	308.105	309.113	0.541	0.981	Linear diaryl-heptanoids	Shikimates and Phenyl-propanoids
14	**Isohypericin**	C_30_H_16_O_8_	504.085	504.084	505.092	−0.191	0.985	Anthra-quinones and anthrones	Polyketides
15	**Arnebinol**	C_16_H_20_O_2_	244.146	244.146	245.154	0.050	1.012	Meromono-terpenoids	Terpenoids
16	**Lusianthridin**	C_15_H_14_O_3_	242.094	242.095	243.102	0.340	1.252	Phenanth-renes	Shikimates and Phenyl-propanoids
17	**Epigallocatechin(4beta,8)-gallocatechin**	C_30_H_26_O_14_	610.132	610.135	611.142	2.787	1.310	Pro-anthocyanins	Shikimates and Phenyl-propanoids
18	**7-O-Methylmorroniside**	C_18_H_28_O_11_	420.163	420.161	421.169	−1.694	1.350	Iridoids monoterpenoids	Terpenoids
19	**Flazin**	C_17_H_12_N_2_O_4_	308.080	308.078	309.086	−1.211	1.490	Carboline alkaloids	Alkaloids
20	**3-O-Methylellagic acid**	C_15_H_8_O_8_	316.022	316.022	317.030	0.394	1.763	Gallotannins	Shikimates and Phenyl-propanoids
21	**8-Hydroxydihydro-chelerythrine**	C_21_H_19_NO_5_	365.126	365.128	366.135	1.299	5.614	Isoquinoline alkaloids	Alkaloids
22	**Eupalinolide A**	C_24_H_30_O_9_	462.189	462.190	463.198	1.322	7.124	Germacrane sesqui-terpenoids	Terpenoids
23	**Erysimoside**	C_35_H_52_O_14_	696.336	696.337	697.344	1.390	7.127	Cardenolides	Terpenoids
24	**Taurocholic acid**	C_26_H_45_NO_7_S	515.292	515.294	516.301	2.086	7.129	Cholane steroids	Terpenoids
25	**Ganoderenic acid A**	C_30_H_42_O_7_	514.293	514.291	515.299	−1.804	7.129	Lanostane, Tirucallane and Euphane triterpenoids	Terpenoids
26	**Lappaol H**	C_40_H_46_O_14_	750.289	750.292	751.299	2.987	7.132	Dibenzyl-butyrolactone lignans	Shikimates and Phenyl-propanoids
27	**Capnoidine**	C_20_H_17_NO_6_	367.106	367.105	368.112	−1.017	7.133	Isoquinoline alkaloids	Alkaloids
28	**Kansuinin E**	C_41_H_47_NO_14_	777.300	777.296	778.303	−3.891	7.133	Jatrophane diterpenoids	Terpenoids
29	**Ganoderic acid B**	C_30_H_44_O_7_	516.309	516.307	517.314	−2.202	7.666	Lanostane, Tirucallane and Euphane triterpenoids	Terpenoids
30	**Tenuifolin**	C_36_H_56_O_12_	680.377	680.379	681.386	1.986	7.685	Oleanane triterpenoids	Terpenoids
31	**N,N′-Dicarbazyl**	C_24_H_16_N_2_	332.131	332.131	333.139	−0.004	7.685	Carboline alkaloids	Alkaloids
32	**Cichorioside B**	C_21_H_28_O_10_	440.168	440.170	441.177	1.442	7.774	Guaiane sesqui-terpenoids	Terpenoids
33	**Daturametelin I**	C_34_H_48_O_10_	616.325	616.322	617.330	−2.294	7.917	Ergostane steroids	Terpenoids
34	**6alpha-Acetoxy-5-epilimonin**	C_28_H_32_O_10_	542.252	542.253	543.260	0.973	7.926	Limonoids	Terpenoids
35	**Pulchinenoside B**	C_53_H_86_O_22_	1074.561	1074.563	1075.570	1.826	7.928	Lupane triterpenoids	Terpenoids
36	**Lactucin**	C_15_H_16_O_5_	276.100	276.100	277.108	0.511	7.930	Guaiane sesqui-terpenoids	Terpenoids
37	**Andrographatoside**	C_26_H_42_O_9_	498.283	498.285	499.292	2.279	7.931	Labdane diterpenoids	Terpenoids
38	**Agrimol A**	C_37_H_46_O_12_	682.299	682.299	683.306	0.090	8.136	Oligomeric phlorogluci-nols (phloro-tannins)	Polyketides
39	**Nimbolidin C**	C_37_H_50_O_12_	686.330	686.331	687.339	1.031	8.138	Limonoids	Terpenoids
40	**Curcumadiol**	C_15_H_26_O_2_	238.193	238.194	239.201	0.912	8.140	Guaiane sesqui-terpenoids	Terpenoids
41	**Evoprenine**	C_20_H_21_NO_4_	339.147	339.146	340.153	−0.924	8.141	Acridone alkaloids	Alkaloids
42	**6-Aldehydo-7-methoxyiso-ophiopogonanone B**	C_20_H_16_O_7_	354.110	354.112	355.119	1.242	8.143	Chromones	Polyketides
43	**Galloylpaeoniflorin**	C_30_H_32_O_15_	632.174	632.176	633.183	2.065	8.161	Pinane monoterpenoids	Terpenoids
44	**Wuweizi alcohol A**	C_24_H_32_O_7_	432.215	432.214	433.222	−0.402	8.300	Dibenzo-cycloocta-dienes lignans	Shikimates and Phenyl-propanoids
45	**Kirenol**	C_20_H_34_O_4_	338.246	338.247	339.255	1.573	8.393	Pimarane and Isopimarane diterpenoids	Terpenoids
46	**Cyclocurcumin**	C_21_H_20_O_6_	368.126	368.127	369.134	1.210	8.394	Linear diarylheptanoids	Shikimates and Phenyl-propanoids
47	**Magnaldehyde B**	C_18_H_16_O_3_	280.110	280.110	281.117	−0.336	8.550	Neolignans	Shikimates and Phenyl-propanoids
48	**Ganoderic acid gamma**	C_30_H_44_O_7_	516.309	516.307	517.314	−1.645	8.592	Lanostane, Tirucallane and Euphane triterpenoids	Terpenoids
49	**Ganoderic acid alpha**	C_32_H_46_O_9_	574.314	574.317	575.324	2.524	8.938	Lanostane, Tirucallane and Euphane triterpenoids	Terpenoids
50	**Licoflavone A**	C_20_H_18_O_4_	322.121	322.120	323.128	−0.212	8.961	Flavones	Shikimates and Phenyl-propanoids
51	**1-Methyl-2-undecyl-4(1H)-quinolone**	C_21_H_31_NO	313.241	313.239	314.247	−1.283	9.247	Quinoline alkaloids	Alkaloids
52	**Stigmast-4-ene-3,6-dione**	C_29_H_46_O_2_	426.350	426.348	427.355	−2.134	9.275	Stigmastane steroids	Terpenoids
53	**Belladonnine**	C_34_H_42_N_2_O_4_	542.314	542.316	543.324	1.775	9.477	Tropane alkaloids	Alkaloids
54	**1-Methyl-2-[(Z)-7-tridecenyl]-4(1H)-quinolone**	C_23_H_33_NO	339.256	339.255	340.262	−1.356	9.488	Quinoline alkaloids	Alkaloids
55	**Meranzin hydrate**	C_15_H_18_O_5_	278.115	278.116	279.123	0.542	9.489	Simple coumarins	Shikimates and Phenyl-propanoids
56	**Cissogenin**	C_21_H_34_O_5_	366.241	366.240	367.247	−0.818	9.617	Pregnane steroids	Terpenoids
57	**Phytolaccagenin**	C_31_H_48_O_7_	532.340	532.339	533.346	−1.012	9.638	Oleanane triterpenoids	Terpenoids
58	**Ajugasterone C-2,3,20,22-diacetonide**	C_33_H_52_O_7_	560.371	560.374	561.381	2.279	9.654	Ecdysteroids	Terpenoids
59	**Sophoranodichromane A**	C_26_H_30_O_5_	424.189	424.189	425.196	0.267	9.682	Flavanones	Shikimates and Phenyl-propanoids
60	**Pterodontoside H**	C_21_H_36_O_7_	400.246	400.247	401.254	1.029	9.830	Eudesmane sesqui-terpenoids	Terpenoids
61	**Picrasinoside G**	C_28_H_44_O_12_	572.283	572.286	573.293	2.630	9.905	Quassinoids	Terpenoids
62	**Nomilin**	C_28_H_34_O_9_	514.220	514.222	515.230	1.945	10.139	Limonoids	Terpenoids
63	**Cinobufagin**	C_26_H_34_O_6_	442.236	442.238	443.245	2.011	10.154	Bufa-dienolides	Terpenoids
64	**Gentianamine**	C_11_H_11_NO_3_	205.074	205.074	206.081	−0.361	10.219	Pyridine alkaloids	Alkaloids
65	**Neohecogenin-3-O-beta-D-glucopyranoside**	C_33_H_52_O_9_	592.361	592.364	593.371	2.804	10.234	Spirostane steroids	Terpenoids
66	**11-Hydroxy-9-tridecenoic acid**	C_13_H_24_O_3_	228.173	228.174	229.181	1.140	10.473	Hydroxy fatty acids	Fatty acids
67	**Cucurbitacin B**	C_32_H_46_O_8_	558.319	558.317	559.324	−2.494	10.646	Cucurbitane triterpenoids	Terpenoids
68	**Clinopodiside F**	C_49_H_82_O_20_	990.540	990.540	991.547	−0.099	10.756	Oleanane triterpenoids	Terpenoids
69	**Daturametelin D**	C_29_H_40_O_5_	436.261	436.262	437.270	0.932	10.834	Ergostane steroids	Terpenoids
70	**Marsdenoside B**	C_45_H_68_O_14_	832.461	832.462	833.469	1.019	10.896	Pregnane steroids	Terpenoids
71	**24-Acetate alisol F**	C_32_H_50_O_6_	530.361	530.362	531.369	1.157	10.920	Fusidane triterpenoids	Terpenoids
72	**Agroastragaloside IV**	C_49_H_80_O_20_	988.524	988.521	989.528	−3.627	10.964	Cucurbitane triterpenoids	Terpenoids
73	**Syringaresinolmono-beta-D-glucoside**	C_24_H_28_O_10_	592.252	592.251	593.258	−0.973	11.109	Furofuranoid lignans	Shikimates and Phenyl-propanoids
74	**Blestrianol B**	C_37_H_32_O_7_	588.215	588.218	589.225	2.909	11.115	Phenanth-renes	Shikimates and Phenyl-propanoids
75	**Brucine**	C_23_H_26_N_2_O_4_	394.189	394.188	395.195	−1.426	11.535	Aspidosperma-Iboga hybrid type (Vinca alkaloids)	Alkaloids
76	**Soyasaponin III**	C_42_H_68_O_14_	812.492	812.495	813.502	2.725	11.538	Oleanane triterpenoids	Terpenoids
77	**Huangqiyenin B**	C_36_H_60_O_10_	652.419	652.417	653.425	−1.313	11.580	Oleanane triterpenoids	Terpenoids
78	**Lappaol B**	C_31_H_34_O_9_	550.220	550.222	551.229	1.576	11.593	Dibenzyl-butyrolactone lignans	Shikimates and Phenyl-propanoids
79	**Curculigo saponin A**	C_36_H_60_O_9_	636.424	636.424	637.431	0.346	11.605	Cycloartane triterpenoids	Terpenoids
80	**Interiotherin C**	C_30_H_36_O_10_	556.231	556.231	557.238	−0.229	11.614	Dibenzo-cycloocta-dienes lignans	Shikimates and Phenyl-propanoids
81	**Simalikalactone D**	C_25_H_34_O_9_	478.220	478.219	479.226	−1.136	11.677	Quassinoids	Terpenoids
82	**Gypenoside XVII**	C_48_H_82_O_18_	946.550	946.546	947.553	−4.017	11.912	Dammarane and Protostane triterpenoids	Terpenoids
83	**Icariin**	C_33_H_40_O_15_	676.237	676.233	677.241	−3.292	12.025	Flavonols	Shikimates and Phenyl-propanoids
84	**Cistanoside C**	C_31_H_38_O_15_	638.221	638.223	639.231	2.404	12.027	Cinnamic acids and derivatives	Shikimates and Phenyl-propanoids
85	**Carmichaeline**	C_22_H_35_NO_4_	377.257	377.255	378.262	−1.437	12.160	Terpenoid alkaloids	Alkaloids
86	**Tenacissoside L**	C_42_H_72_O_16_	832.482	832.480	833.487	−2.339	12.208	Pregnane steroids	Terpenoids
87	**Hordatine B**	C_29_H_40_N_8_O_5_	580.312	580.311	581.318	−1.252	12.315	Neolignans	Alkaloids
88	**Tsugaric acid B**	C_33_H_52_O_5_	528.381	528.382	529.389	0.577	12.364	Lanostane, Tirucallane and Euphane triterpenoids	Terpenoids
89	**Lablaboside C**	C_60_H_96_O_28_	1264.609	1264.610	1265.617	1.158	12.403	Oleanane triterpenoids	Terpenoids
90	**Vaccaroside B**	C_60_H_94_O_29_	1278.588	1278.582	1279.589	−6.100	12.406	Oleanane triterpenoids	Terpenoids
91	**Pingpeimine A**	C_27_H_45_NO_5_	463.330	463.331	464.339	1.542	12.421	Steroidal alkaloids	Alkaloids
92	**Delphatine**	C_26_H_43_NO_7_	481.304	481.303	482.310	−1.333	12.441	Terpenoid alkaloids	Alkaloids
93	**Neferine**	C_38_H_44_N_2_O_6_	624.320	624.317	625.324	−2.932	12.737	Isoquinoline alkaloids	Alkaloids
94	**Ephedradine B**	C_29_H_38_N_4_O_5_	522.284	522.286	523.293	1.678	12.784	Polyamines	Alkaloids
95	**Adhyperforin**	C_36_H_54_O_4_	550.402	550.401	551.409	−0.932	12.819	Poly-prenylated cyclic polyketides (Hop mero-terpenoids)	Polyketides
96	**Senbusine B**	C_23_H_37_NO_6_	423.262	423.261	424.268	−0.990	12.842	Terpenoid alkaloids	Alkaloids
97	**Sipeimine**	C_27_H_43_NO_3_	429.324	429.326	430.333	1.365	13.053	Steroidal alkaloids	Alkaloids
98	**Lyciumin A**	C_42_H_51_N_9_O_12_	873.366	873.365	874.372	−0.695	13.063	Cyclic peptides	Amino acids and Peptides
99	**Yemuoside YM14**	C_58_H_92_O_25_	1042.535	1042.532	1043.539	−3.317	13.149	Oleanane triterpenoids	Terpenoids
100	**Phytolaccagenic acid**	C_31_H_48_O_6_	516.345	516.346	517.354	1.306	13.305	Oleanane triterpenoids	Terpenoids
101	**Ganolactone**	C_27_H_36_O_6_	456.251	456.249	457.257	−1.749	13.700	Lanostane, Tirucallane and Euphane triterpenoids	Terpenoids
102	**Delbrusine**	C_27_H_43_NO_7_	493.304	493.305	494.312	0.813	13.710	Terpenoid alkaloids	Alkaloids
103	**Tigloylgomisin H**	C_28_H_36_O_8_	500.241	500.243	501.250	1.823	14.015	Dibenzo-cycloocta-dienes lignans	Shikimates and Phenyl-propanoids
104	**Protostemotinine**	C_23_H_29_NO_6_	415.199	415.199	416.206	−0.360	14.544	Stemona alkaloids	Alkaloids
105	**Sanggenon C**	C_40_H_36_O_12_	708.221	708.220	709.228	−0.400	14.911	Chalcones	Shikimates and Phenyl-propanoids
106	**Physanol A**	C_36_H_50_O_4_	546.371	546.371	547.379	0.573	15.226	Ecdysteroids	Terpenoids
107	**Cumambrin A**	C_17_H_22_O_5_	306.147	306.146	307.153	−1.140	15.798	Guaiane sesqui-terpenoids	Terpenoids
108	**Physanol B**	C_36_H_52_O_4_	548.387	548.387	549.394	0.310	16.221	Ecdysteroids	Terpenoids
109	**Daturametelin C**	C_29_H_40_O_5_	468.288	468.290	469.297	2.253	16.243	Ergostane steroids	Terpenoids
110	**Aurapten**	C_19_H_22_O_3_	298.157	298.157	299.164	0.074	16.261	Simple coumarins	Shikimates and Phenyl-propanoids
111	**Marsdenoside C**	C_47_H_68_O_14_	856.461	856.458	857.465	−3.152	16.275	Pregnane steroids	Terpenoids
112	**Phytolacca cerebroside**	C_48_H_93_NO_10_	843.680	843.678	844.685	−2.314	16.359	Neutral glyco-sphingolipids	Fatty acids
113	**Picroside III**	C_25_H_30_O_13_	538.169	538.169	539.176	0.424	16.508	Iridoids mono-terpenoids	Terpenoids
114	**Isoaloeresin A**	C_28_H_28_O_11_	540.163	540.165	541.172	1.373	16.509	Chromones	Polyketides
115	**1-Vinyl-4-methoxy-beta-carboline**	C_14_H_12_N_2_O	224.095	224.095	225.102	−0.297	16.529	Carboline alkaloids	Alkaloids
116	**Kushenol B**	C_30_H_36_O_6_	492.251	492.253	493.260	1.656	16.556	Flavanones	Shikimates and Phenyl-propanoids
117	**Asterinin A**	C_25_H_33_N_5_O_8_	531.233	531.235	532.242	1.720	16.784	Tripeptides	Amino acids and Peptides
118	**Marmesinin**	C_20_H_24_O_9_	408.142	408.144	409.151	1.991	16.867	Furo-coumarins	Shikimates and Phenyl-propanoids

#### Anti-proliferation activity of *n*-butanol fraction.

The antiproliferative activity of the ethanol fraction (crude extract), n-butanol, and Dichlorometane fraction fraction was evaluated against three cancer cell lines, including breast cancer (MCF-7) and gastric cancer (MKN45 and AGS), using the MTT assay. The half-maximal inhibitory concentration (IC_50_) values of the extracts and fractions shown in Table 3 (and see more in S2 Table, S3 Table, and S4 Table) indicate that the effects on cell proliferation differed among the crude extract (ethanol extract) and fractions, as well as among the tested cell lines. The *n*-butanol fraction exhibited the strongest activity, showing low IC_50_ values with narrow confidence intervals in the evaluated cell lines, particularly in AGS cells (51.7 µg/mL). In contrast, the ethanol fraction showed the weakest activity, as reflected by higher IC_50_ values, most notably in AGS cells (167.4 µg/mL). Among the cell lines, AGS appeared more sensitive than MCF7 and MKN45 to the tested fractions. Therefore, the *n*-butanol fraction effect in AGS cells was selected for subsequent mechanistic investigations and more analyses.

**Table 3:** Effects of *n*-butanol fraction on the proliferation of cancer cells.

Cell line	Ethanol fraction (crude extract)	Dichloromethane fraction	*n*-Butanol fraction	Overall p-value
MCF7	79.3 (63.8–97.4)	60.5 (49.5–71.9)	60.2 (55.1–65.8)	< 0.05
MKN45	—	105.1 (85.9–127.6)	85.2 (73.5–98.7)	> 0.05
AGS	167.4 (138.6–204.3)	80.8 (56.8–110.5)	51.7 (45.1–58.3)	< 0.05

*Data are presented as IC50 (95% confidence interval). Overall differences among treatments were assessed by one-way ANOVA.*

**Fig 3:**
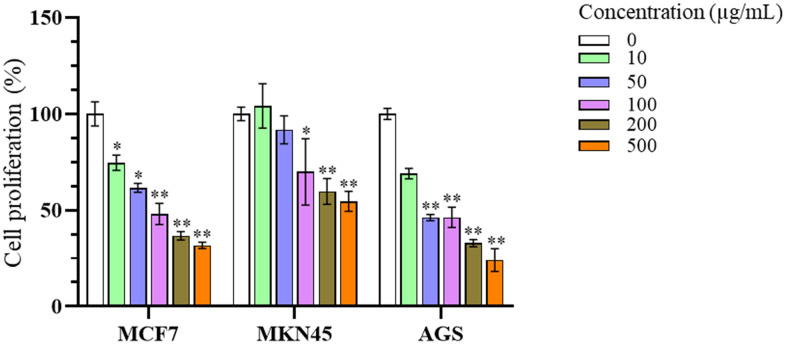
Effect of *n*-butanol fraction on cell proliferation of cancer cell lines. Cells were treated with the *n*-butanol fraction at various concentrations (0–500 µg/mL) for 48 h and subsequently analyzed using the MTT assay. Data are presented as mean ± SD. Statistical significance was assessed using the Mann–Whitney U test: p < 0.05, p < 0.01, and p < 0.001.

As shown in Fig 3, the cell viability of all tested cell lines decreased in a dose-dependent manner across concentrations ranging from 0 µg/mL to 500 µg/mL. Even at the initial concentration of 10 µg/mL, a reduction in proliferation was observed, with MCF-7 and AGS cell viability decreasing to 74.6 ± 3.9% and 69.0 ± 2.7%, respectively. Notably, significant growth inhibition was recorded at concentrations of 100 µg/mL and above, where MKN45 cell viability dropped to 69.9 ± 17.2% (p < 0.05), while MCF-7 and AGS cell viabilities were markedly reduced to 48.1 ± 5.5% and 46.3 ± 5.2%, respectively (p < 0.01).

### *n*-butanol fraction inhibits tumorsphere formation and growth of AGS gastric cancer cells

**Fig 4:**
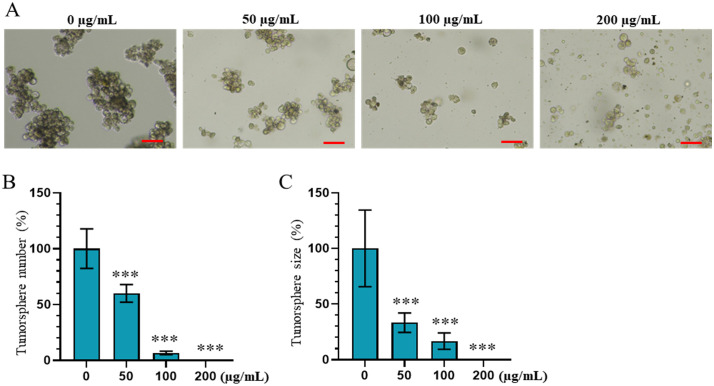
Effects of *n*-butanol fraction on the tumorsphere formation of AGS gastric cancer. **(A)** Effect of fraction on tumorsphere morphology, **(B)** on tumorsphere number, and **(C)** on tumorsphere size. Data are presented as the mean ± standard deviation, Mann-Whitney test, ***p < 0.001 compared to the control; Scale bar = 100 µm.

To assess the invasive potential of cancer cells, a 3D culture system was employed to induce the formation of tumorspheres, a characteristic closely associated with tumor maintenance and progression. The effects of the *n*-butanol fraction on tumorsphere formation are illustrated in Fig 4, with Fig 4C highlighting the impact of the extract on tumorsphere size. The results demonstrate that both the number and size of tumorspheres decreased progressively with increasing concentrations of the extract (50–200 µg/mL). At a concentration of 50 µg/mL, a significant reduction in both tumorsphere number and size was observed compared to the control (p < 0.01). Upon increasing the concentration to 100 µg/mL, the number of tumorspheres dropped to approximately 5%, and their size was reduced to about 15% relative to the untreated group. Notably, at the highest tested concentration of 200 µg/mL, tumorsphere formation was almost completely inhibited. These findings clearly indicate that under 3D culture conditions, the *n*-butanol fraction exerts a pronounced inhibitory effect on the tumorigenic capacity of cancer cells.

### *n*-butanol fraction reduces migration ability of of AGS gastric cancer cells

**Fig 5:**
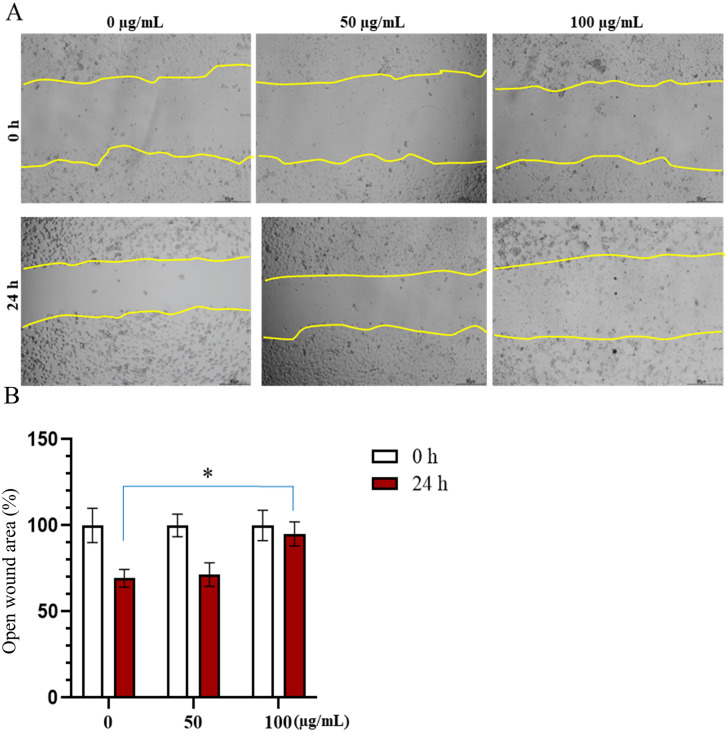
Effect of *n*-butanol fraction on the migration of AGS cells. **(A)** Representative images of wound healing assay showing the migratory ability of AGS cells treated with different concentrations (0, 50 and 100 μg/mL) of *n*-butanol fraction at 0 and 24 hours. The yellow lines delineate the wound edges. **(B)** Quantitative analysis of wound closure percentage at 0 and 24 hours. Data are presented as mean ± standard deviation. Mann-Whitney test, *p < 0.05, **p < 0.01 compared to the control group (0 μg/mL).

The inhibitory effect of the *n*-butanol fraction on AGS gastric cancer cell migration is presented in Fig 5. The results revealed clear differences in cell migration based on the wound boundary observed in control wells compared to those treated with the *n*-butanol fraction. In the control group, AGS cells exhibited strong migratory activity, significantly narrowing the wound area after 24 hours, to approximately 70% of the initial width at 0 hours. At a concentration of 50 µg/mL, the extract did not result in a significant difference in migration compared to the untreated control. However, at a concentration of 100 µg/mL, a marked inhibition of cell migration was observed. Cells showed substantially reduced or complete absence of movement into the wound area, and most of the observed cells were either non-motile or dead. The measured wound widths in the 100 µg/mL treatment groups remained at approximately 95%, respectively, compared to the control group at 24 hours (p < 0.05). These findings suggest that the *n*-butanol fraction impairs the migratory capacity of AGS cells in a dose-dependent manner.

### *n*-butatnol fraction induces cell senescence

**Fig 6:**
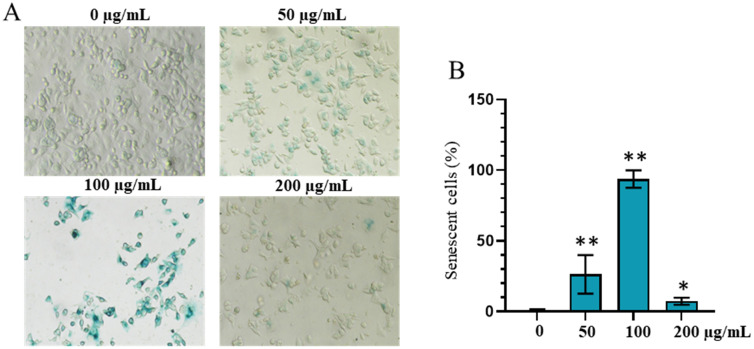
Effect of *n*-butanol fration on cellular senescence in AGS gastric cancer cells. **(A)** Representative images of senescence-associated β-galactosidase (SA-β-gal) staining in AGS cells treated with different concentrations (0, 50, 100, and 200 μg/mL) of *n*-butanol fraction. Senescent cells are stained blue. **(B)** Quantitative analysis of the percentage of SA-β-gal-positive cells. Data are presented as mean ± standard deviation. Mann-Whitney test, *p < 0.05, **p < 0.01 compared to the control (0 μg/mL).

The impact of the *n*-butanol fraction on cellular senescence in AGS cells is presented in Fig 6. Treatment with 50 µg/mL of the extract resulted in a marked increase in senescent cell number—approximately 25-fold compared to the control group (p < 0.01). At a higher concentration of 100 µg/mL, the extract induced the most pronounced senescence, as evidenced by a substantial accumulation of SA-β-gal-positive cells, showing an approximately 93-fold increase relative to the control (p < 0.01). Interestingly, at the highest concentration tested (200 µg/mL), the proportion of senescent cells significantly decreased compared to the 50 and 100 µg/mL treatments. Instead, a notable increase in cell death was observed, indicating a shift in the cellular response from senescence induction to cytotoxicity (p < 0.05).

### *n*-butanol fraction induces cell cycle arrest at G0/G1 phase

**Fig 7:**
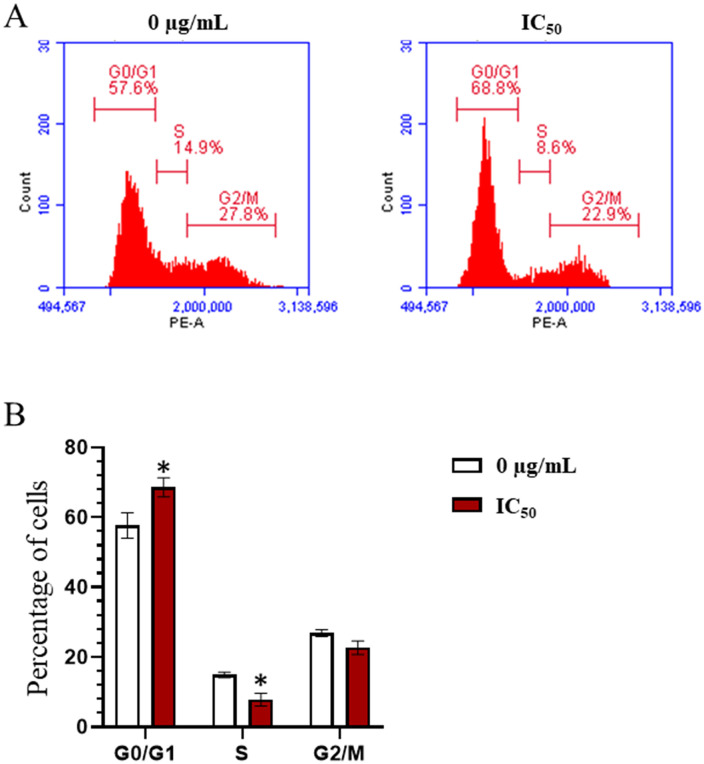
Effect of *n*-butanol fraction on AGS cell cycle. **(A)** Cell cycle analysis plots by flow cytometry showing the percentage of AGS cells in different phases after treatment with *n*-butanol fraction and the control group. **(B)** Quantitative analysis of the percentage of cells in G0/G1, S, and G2/M phases. Data are presented as the mean ± standard deviation, Mann-Whitney test, *p < 0.05.

The effects of the *n*-butanol fraction on the cell cycle were evaluated by flow cytometry analysis. Cell cycle profiling (Fig 7) revealed that the *n*-butanol fraction induced cell cycle arrest in AGS cells. Specifically, there was an accumulation of cells in the G0/G1 phase in the *n*-butanol-treated group (68.6 ± 2.7%) compared to the control group (57.7 ± 3.6%), respectively (p < 0.05). This was accompanied by a reduction in the S phase population (p < 0.05), indicating that the extract effectively induced G0/G1 phase arrest.

**Fig 8:**
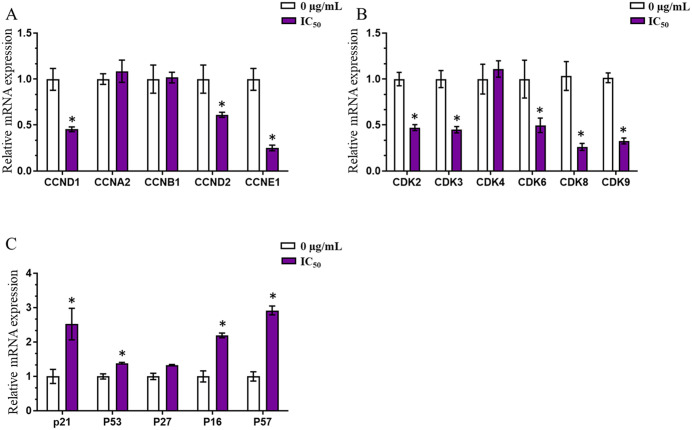
Effect of *n*-butanol fraction on cell cycle regulatory gene expression at mRNA level in AGS gastric cancer cells. Cells were treated with *n*-butanol fraction at concentration of IC_50_ (52.7 µg/mL) for 24 hours. RNA total was extracted for realtime PCR analysis. Changes in mRNA levels of genes were analysis by 2^–∆∆Ct^ method. **(A)** Bar graphs showing the expression of genes belonging to the cyclin group, **(B)** genes belonging to the cyclin-dependent kinase group, and **(C)** genes belonging to the cyclin inhibitor group. Data are presented as the mean ± standard deviation. All results are presented as mean ± SD (n = 3). Mann-Whitney test, *p < 0.05 compared to control (0 µg/mL).

Real-time PCR analysis (Fig 8) showed that the *n*-butanol fraction at the IC_50_ concentration markedly altered the expression of cell cycle–related genes compared with the 0 µg/mL control. The *n*-butanol fraction significantly downregulated pro-proliferative cyclin genes, including CCND1, CCND2, and CCNE1, whereas CCNA2 and CCNB1 exhibited only minor or no apparent changes. In parallel, the expression of CDK family genes (CDK2, CDK3, CDK6, CDK8, and CDK9) was significantly reduced. Conversely, the *n*-butanol fraction significantly upregulated cell cycle inhibitory and anti-proliferative response genes such as P21, P53, P16, and P57, while P27 remained largely unchanged. Collectively, these data indicate that the *n*-butanol fraction suppresses cell proliferation primarily by inhibiting the Cyclin–CDK axis and activating cell cycle inhibitory signaling pathways.

#### Molecular docking.

**Fig 9:**
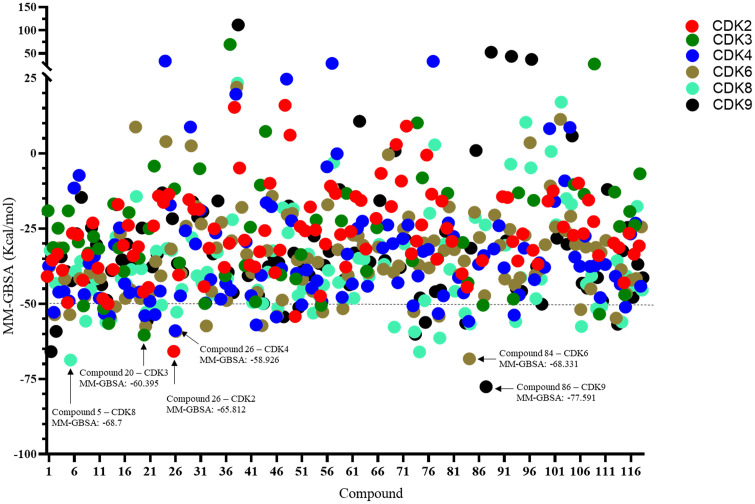
Molecular docking analysis results of compounds from the *n*-butanol fraction with cyclin-dependent proteins.

To evaluate the reliability of the docking protocol, re-docking experiments were conducted in which co-crystallized ligands were redocked into the active sites of their respective target proteins. In all cases, the resulting RMSD values were below 2.0 Å, indicating high reproducibility and accuracy of the docking methodology employed in this study. A total of 118 compounds identified from *Ardisia villosa n*-butanol fraction were subjected to virtual screening against six cell cycle-regulating cyclin-dependent kinases (CDK2, CDK3, CDK4, CDK6, CDK8, and CDK9) using the Glide XP, followed by rescoring with Prime MM-GBSA to refine binding energy estimates. A comprehensive overview of the MM-GBSA results is presented in Fig 9. Each data point corresponds to a ligand–protein complex, with the x-axis indicating compound index (from 1 to 118), the y-axis representing MM-GBSA values (kcal/mol), and color-coding distinguishing among the six CDK targets. This visualization facilitates the comparative analysis of binding affinity distributions across targets.

The majority of ligands displayed binding free energies in the range of −25 to −50 kcal/mol. Specifically, the number of compounds within this range were: CDK2 (59 compounds), CDK3 (26), CDK4 (67), CDK6 (72), CDK8 (63), and CDK9 (65). Notably, the proportion of compounds exhibiting ΔG_bind_ values below −50 kcal/mol ranged from 2.54% to 17.8%, depending on the target. CDK8 had the highest number of strong binders (21 compounds), while CDK2 had the fewest (only 3 compounds).

**Fig 10:**
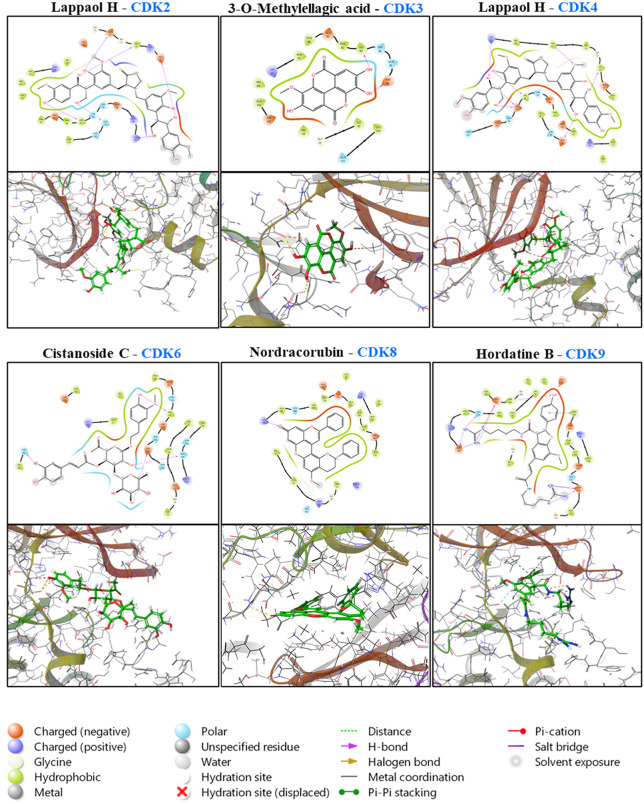
2D and 3D interaction diagrams of the compounds with the target proteins exhibiting the highest binding energies.

From this dataset, the top-scoring ligand for each CDK was identified based on the lowest ΔGbind score. Compound 26 emerged as the strongest binder for both CDK2 (−65.812 kcal/mol) and CDK4 (−58.926 kcal/mol). The highest-affinity ligands for the remaining targets were: compound 20 for CDK3 (−60.395 kcal/mol), compound 84 for CDK6 (−68.331 kcal/mol), compound 5 for CDK8 (−68.7 kcal/mol), and compound 87 for CDK9 (−77.591 kcal/mol). A detailed summary of binding affinities and molecular interaction profiles for these ligand–target complexes is provided in Table 4. Fig 10 illustrates representative 2D and 3D spatial interactions of each ligand within the active site of its corresponding protein.

**Table 4:** Docking Scores, and MM-GBSA energies of compounds from *n*-butanol fraction with target CDK proteins.

Protein	Ligand(compound)	Docking score (kcal/mol)	MM-GBSA (kcal/mol)	Hydrophilic	Hydrophobic	Electrostatic
**CDK2**	**Lappaol H**	−10.681	−65.812	LYS89, GLU8, GLU12, ILE10, GLN131,LEU8, LYS33, HIS84, GLU162, LYS9	ALA31, ILE10, LYS9, VAL64, LEU134, VAL18	GLU8
**CDK3**	**3-O-Methylellagic acid**	−7.952	−60.395	LYS22, ASP97, GLU144,ASN145, VAL14, GLU11	ALA10, VAL72, LYS22	
**CDK4**	**Lappaol H**	−10.311	−58.926	LYS147, GLN149, ASP104, VAL101, THR182, HIS100, LYS147, GLN149, TYR24	ILE19, LEU152, ALA41	
**CDK6**	**Cistanoside C**	−10.306	−68.331	LYS147, GLN149, ASP104, VAL101, THR182, HIS100, TYR24	ILE19, LEU152, ALA41	
**CDK8**	**Nordracorubin**	−6.515	−68.7	LYS153	TYR32, VAL27, VAL35, ALA50, ALA100, LEU158, LYS52, ALA172	
**CDK9**	**Hordatine B**	−9.573	−77.591	ASP167, ASP109, PHE168, GLU66, ASP149, ASN154, PHE105, ASP154, ASP104, GLU107	PHE103, ALA46, VAL79, CYS106, LEU156, ILE25, ALA46, ALA166	ASP167, ASP109, ASP149

## Discussion

Accurate identification of the chemical constituents present in plant extracts is critically important for predicting and evaluating their potential biological activities. In this study, ultra-performance liquid chromatography coupled with quadrupole time-of-flight mass spectrometry (UPLC-QToF-MS) was employed for the chemical profiling of the *n*-butanol fraction obtained from *Ardisia villosa* leaves. This analytical technique offers high sensitivity and precision; a signal intensity threshold of >5000 counts and a mass error tolerance of 5 ppm were applied to ensure the reliable detection and identification of constituents in the extract.

Mass spectrometric analysis revealed a diverse array of metabolites, with a total of 118 compounds identified. These compounds were classified into various chemical categories, including triterpenoids (37 compounds), alkaloids (22 compounds), flavonoids (10 compounds), lignans (10 compounds), steroids (13 compounds), coumarins (5 compounds), phenolic and aromatic compounds (12 compounds), along with other functional constituents. Notably, there has been limited prior research on the phytochemical composition of *Ardisia villosa*, and thus, the majority of the 118 identified compounds are reported here for the first time, made possible by the high sensitivity of the UPLC-QToF-MS platform.

Significantly, numerous compounds identified and enumerated in Table 1 have been previously documented to demonstrate anticancer activity across various cancer cell lines. For example, within the flavonoid and phenolic groups, notable compounds include ononin (compound 3) [22], epigallocatechin-(4β → 8)-gallocatechin (compound 17) [23], icariin (compound 83) [24], and sanggenon C (compound 105) [25]. Among the terpenoids and steroids, compounds such as bruceene (6), ganoderenic acid A (25) [26], cucurbitacin B (67) [27], and simalikalactone D (81) [28] were identified. In the alkaloid category, brucine (75) [29], Pingpeimine A (91) [30], và Neferine (93) [31] were detected.

The anticancer activity of the *n*-butanol fraction was evaluated against three different cancer cell lines MCF-7, MKN45, and AGS, and the results demonstrated that the extract exhibited the highest sensitivity toward AGS cells, with an IC_50_ value of 51.7 ± 2.8 µg/mL. Previous cytotoxicity studies on other *Ardisia* species reported that an ethanol extract from *Ardisia gigantifolia* exerted inhibitory effects on gastric cancer cell lines MKN45 and MKN74, with IC_50_ values of 55.7 and 123.6 µg/mL, respectively [12]. In addition, earlier investigations revealed that the *n*-butanol fraction of *Pterocephalus hookeri* displayed cytotoxic effects on the Hep3B liver cancer cell line (IC_50_ = 90.8 µg/mL) and Caco-2 colorectal cancer cells (IC_50_ = 175.2 µg/mL) [32].

These findings suggest that the antiproliferative potency of plant extracts can vary significantly depending on both the plant species and the extraction solvent used. Differences in solvent polarity and chemical affinity may lead to the enrichment of distinct sets of bioactive compounds, which ultimately influence the biological activity of the extracts.

In recent years, the 3D tumorsphere model has been considered superior to conventional 2D culture systems for toxicity testing due to its high physiological relevance to in vivo tumors. This model allows for more accurate evaluation of drug efficacy and enhances the screening potential for novel therapeutic agents [33,34]. In this study, the *n*-butanol fraction markedly inhibited tumorsphere formation in AGS gastric cancer cells, underscoring its broad-spectrum anticancer potential. The observed reduction in both the number and size of tumorspheres suggests that bioactive compounds in this fraction effectively target even highly proliferative subpopulations within the tumorspheres.

Cells capable of forming tumorspheres have previously been shown to exhibit cancer stem cell-like properties [34], implying that the butanol fraction may modulate critical signaling pathways essential for cancer stem cell survival and maintenance. Further studies are warranted to elucidate these underlying mechanisms. Cell migration is a key indicator of tumor cell viability, invasiveness, and metastatic potential [34]. In the present study, migration analysis revealed that the n-butanol fraction significantly suppressed the ability of AGS cancer cells to migrate into the wound area created on the culture plate surface. These findings are consistent with earlier results showing that the *n*-butanol fraction reduced AGS cell proliferation and are also in agreement with previous studies reporting anticancer activity of *n*-butanol fraction from *Ricinus communis* in MCF-7 breast cancer cells [35], and the Wenxia Formula Extract in H460 and A549 lung cancer cell lines [36].

Cellular senescence is increasingly recognized as a promising strategy in the development of next-generation cancer therapies. By shifting cancer cells from an uncontrolled proliferative state to an irreversible growth arrest, senescence can effectively impede tumor progression [35]. A key objective of this study was to investigate whether the *n*-butanol fraction could induce senescence in AGS gastric cancer cells.

Our findings revealed that treatment with the *n*-butanol fraction at concentrations of 50 and 100 µg/mL induced a pronounced senescent phenotype in AGS cells. In contrast, at higher concentrations (e.g., 200 µg/mL), senescent cells were no longer observed, and instead, a significant increase in cell death was detected, suggesting a shift from a senescence-inducing effect to a cytotoxic response. This concentration-dependent relationship between senescence and cell death aligns with established mechanisms in which sub-lethal drug exposure promotes senescence, whereas higher concentrations trigger apoptosis or necrosis [36].

The data indicate that the *n*-butanol fraction can induce either cellular senescence or apoptosis, contingent upon the administered dose. Both mechanisms contribute to the inhibition of cancer cell proliferation and may present a dual-mode therapeutic advantage.

As previously mentioned, cellular senescence is characterized by irreversible cell cycle arrest, often associated with the accumulation of cells in the quiescent G0 phase. This hypothesis was investigated through cell cycle analysis (Fig 7). A notable increase in the proportion of cells in the G0/G1 phase compared to the control group indicates that the *n*-butanol fraction delays cell cycle progression—a critical mechanism contributing to senescence induction.

Further validation using real-time PCR analysis revealed a marked downregulation of several key genes involved in cell cycle progression, particularly those belonging to the cyclin family (CCND1, CCND2, CCNE1) and cyclin-dependent kinases (CDK2, CDK3, CDK6, CDK8, and CDK9), all of which play essential roles in regulating cell proliferation and oncogenesis As previously mentioned, cellular senescence is characterized by irreversible cell cycle arrest, often associated with the accumulation of cells in the quiescent G0 phase. This hypothesis was investigated through cell cycle analysis (Fig 7). A notable increase in the proportion of cells in the G0/G1 phase compared to the control group indicates that the *n*-butanol fraction delays cell cycle progression—a critical mechanism contributing to senescence induction.

Further validation using real-time PCR analysis revealed a marked downregulation of several key genes involved in cell cycle progression, particularly those belonging to the cyclin family (CCND1, CCND2, CCNE1) and cyclin-dependent kinases (CDK2, CDK3, CDK6, CDK8, and CDK9), all of which play essential roles in regulating cell proliferation and oncogenesis [37].

Remarkably, genes encoding major negative regulators of the cell cycle and established markers of cellular senescence, including p21, p53, p16, and p57, were significantly upregulated following treatment [38,39]. These findings indicate that the *n*-butanol fraction modulates the expression of both pro-proliferative and anti-proliferative genes, thereby promoting cell cycle arrest and senescence in AGS gastric cancer cells.

In addition to the observed transcriptional downregulation of cyclin and CDK genes, we hypothesized that certain compounds present in the *n*-butanol fraction of *Ardisia villosa* may directly inhibit the activity of CDK proteins through high-affinity binding to their active sites. Based on MM-GBSA binding free energy values obtained from in silico molecular docking analyses, several compounds within the extract demonstrated strong potential for CDK inhibition, with binding energies below –50 kcal/mol. Notably, compounds such as Lappaol H, 3-O-Methylellagic acid, Cistanoside C, Nordracorubin, and Hordatine B exhibited the strongest binding affinities toward CDK family proteins, including CDK2, CDK3, CDK4, CDK6, CDK8, and CDK9.

Previous experimental studies have reported that a compound from the Lappaol family, Lappaol F, induces cell cycle arrest at the S phase in colorectal cancer cells [40], and at the G1 and G2 phases in other cancer cell lines [37]. While experimental evidence supporting the cell cycle inhibitory activity of the other identified compounds is currently lacking, their high binding affinity to CDK proteins strongly suggests that they may serve as promising CDK inhibitors. These findings highlight the potential of these natural compounds as candidates for further investigation in cell-based assays to validate their anticancer mechanisms. However, the limitation of this study is that the biological activity assessments were mainly conducted through in vitro experiments, without in vivo validation or isolation of individual bioactive compounds. Moreover, the molecular docking predictions are only theoretical and require further verification through the isolation of pure compounds followed by experimental evaluation of their biological activities in both *in vitro* and *in vivo* models.

## Conclusion

The c of *Ardisia villosa* demonstrates notable anticancer activity through inhibition of cell proliferation, tumorsphere formation, and migration, as well as induction of cellular senescence. Phytochemical analysis revealed a diverse profile of bioactive compounds. These findings support its potential as a natural anticancer agent and warrant further preclinical investigation to elucidate its therapeutic efficacy and underlying mechanisms.

## Supporting information

S1 Table List of primer for Real-time PCR. (DOCX)

S2 Table Effect of the ethanol extract on cancer cell proliferation. (DOCX)

S3 Table Effect of the n-butanol fraction on cancer cell proliferation. (DOCX)

S4 Table Effect of the dichloromethane fraction on cancer cell proliferation. (DOCX)
