# Supplementary material for: Phytochemical profiling and anticancer activity of the n-butanol fraction from Ardisia villosa extract: Inhibition of gastric cancer cell proliferation via cell cycle arrest and senescence induction
Source: PLoS One. 2026 Jan 8;21(1):e0340458. doi: 10.1371/journal.pone.0340458 (PMC12782380; doi:10.1371/journal.pone.0340458)
Supplement: S1 Table — (DOCX) [file pone.0340458.s001.docx]

**S1 Table. List of primer for Real-time PCR**

| Gene names | Primers | Sequences (5’ 🡪 3’) |
| --- | --- | --- |
| CCND1 | CCND1–Forward (F) | TCTACACCGACAACTCCATCCG |
|  | CCND1–Reverse (R) | TCTGGCATTTTGGAGAGGAAGTG |
| CCND2 | CCND2–F | TCTGGCATTTTGGAGAGGAAGTG |
|  | CCND2–R | CTTCCAGTTGCGATCATCGACG |
| CCNB1 | CCNB1–F | GACCTGTGTCAGGCTTTCTCTG |
|  | CCNB1–R | GGTATTTTGGTCTGACTGCTTGC |
| CCNA2 | CCNA2-F | CTCTACACAGTCACGGGACAAAG |
|  | CCNA2-R | CTGTGGTGCTTTGAGGTAGGTC |
| CCNE1 | CCNE1–F | TGTGTCCTGGATGTTGACTGCC |
|  | CCNE1–R | CTCTATGTCGCACCACTGATACC |
| CDK2 | CDK2– F | ATGGATGCCTCTGCTCTCACTG |
|  | CDK2– R | CCCGATGAGAATGGCAGAAAGC |
| CDK3 | CDK3– F | TCGCTGCTCAAGGAACTGAAGC |
|  | CDK3– R | GTCCTGGCTGAGGAACTCAAAC |
| CDK4 | CDK4– F | CCATCAGCACAGTTCGTGAGGT |
|  | CDK4– R | TCAGTTCGGGATGTGGCACAGA |
| CDK6 | CDK6– F | GGATAAAGTTCCAGAGCCTGGAG |
|  | CDK6– R | GCGATGCACTACTCGGTGTGAA |
| CDK8 | CDK8– F | GCTGATAGGAAGGTGTGGCTTC |
|  | CDK8– R | CCGAGGTAACTGAACTGGCTTC |
| CDK9 | CDK9– F | CCATTACAGCCTTGCGGGAGAT |
|  | CDK9– R | CAGCAAGGTCATGCTCGCAGAA |
| P16 | P16– F | CTCGTGCTGATGCTACTGAGGA |
|  | P16– R | GGTCGGCGCAGTTGGGCTCC |
| P27 | P27– F | CTCGTGCTGATGCTACTGAGGA |
|  | P27– R | GGTCGGCGCAGTTGGGCTCC |
| P21 | P21– F | GACCTGTGTCAGGCTTTCTCTG |
|  | P21– R | GGTATTTTGGTCTGACTGCTTGC |
| P57 | P57 – F | AGATCAGCGCCTGAGAAGTCGT |
|  | P57– R | TCGGGGCTCTTTGGGCTCTAAA |
| HPRT1 | HPRT1– F | CATTATGCTGAGGATTTGGAAAGG |
|  | HPRT1– R | CTTGAGCACACAGAGGGCTACA |
