# Supplementary material for: Phytochemical profiling and anticancer activity of the n-butanol fraction from Ardisia villosa extract: Inhibition of gastric cancer cell proliferation via cell cycle arrest and senescence induction
Source: PLoS One. 2026 Jan 8;21(1):e0340458. doi: 10.1371/journal.pone.0340458 (PMC12782380; doi:10.1371/journal.pone.0340458)
Supplement: S2 Table — (DOCX) [file pone.0340458.s002.docx]

**S2 Table. Effect of the ethanol extract on cancer cell proliferation**

| Cell lines | Concentration of ethanol extract | | | | | | IC_50_ values  (95% CI) |
| --- | --- | --- | --- | --- | --- | --- | --- |
|  | 0 µg/mL | 10 µg/mL | 50 µg/mL | 100 µg/mL | 200 µg/mL | 500 µg/mL |  |
| MCF7 | 100 ± 6.4 | 86.5 ± 10.7* | 63.1 ± 13.8* | 43.5 ± 6.6** | 27.2 ± 6.1** | 17.1 ± 6.1** | 79.3 ± 18.0  (63.8 - 97.4) |
| AGS | 100 ± 16.2 | 98.8 ± 8.2 | 83.8 ± 9.5 | 69.7 ± 13.0* | 41.0 ± 7.9** | 19.4 ± 7.0** | 167.4 ± 38.9  (138.6 – 204.3) |
| Note: Cell proliferation values are presented as % Mean ± SD, * p < 0.05, ** p < 0.01, ***p < 0.001 vs. control. T test. | | | | | | |  |
