# Supplementary material for: Phytochemical profiling and anticancer activity of the n-butanol fraction from Ardisia villosa extract: Inhibition of gastric cancer cell proliferation via cell cycle arrest and senescence induction
Source: PLoS One. 2026 Jan 8;21(1):e0340458. doi: 10.1371/journal.pone.0340458 (PMC12782380; doi:10.1371/journal.pone.0340458)
Supplement: S3 Table — (DOCX) [file pone.0340458.s003.docx]

**S3 Table. Effect of the n-butanol fraction on cancer cell proliferation**

| Cell lines | Concentration of n-butanol fraction | | | | | | IC_50_ values  (95% CI) |
| --- | --- | --- | --- | --- | --- | --- | --- |
|  | 0 µg/mL | 10 µg/mL | 50 µg/mL | 100 µg/mL | 200 µg/mL | 500 µg/mL |  |
| MCF7 | 100 ± 6.3 | 74.6 ± 3.9* | 61.6 ± 2.3* | 48.1 ± 5.5** | 36.7 ± 2.2** | 31.8 ± 1.6** | 60.2 ± 1.9  (55.1 - 65.8) |
| MKN45 | 100 ± 3.6 | 104.1 ± 11.6 | 91.7 ± 7.3 | 69.9 ± 17.2* | 59.7 ± 6.7** | 54.6 ± 5.2** | 85.2 ± 7.1  (73.5 - 98.7) |
| AGS | 100 ± 2.9 | 69.0 ± 2.7 | 46.2 ± 1.6** | 46.3 ± 5.2** | 32.9 ± 1.9** | 24.1 ± 5.9** | 51.7 ± 2.8  (45.1 - 58.3) |
| Note: Cell proliferation values are presented as % Mean ± SD, * p < 0.05, ** p < 0.01 vs. control. T test. | | | | | | |  |
