# Supplementary material for: Phytochemical profiling and anticancer activity of the n-butanol fraction from Ardisia villosa extract: Inhibition of gastric cancer cell proliferation via cell cycle arrest and senescence induction
Source: PLoS One. 2026 Jan 8;21(1):e0340458. doi: 10.1371/journal.pone.0340458 (PMC12782380; doi:10.1371/journal.pone.0340458)
Supplement: S4 Table — (DOCX) [file pone.0340458.s004.docx]

**S4 Table. Effect of the dichloromethane fraction on cancer cell proliferation**

| Cell lines | Concentration of dichloromethane fraction | | | | | | IC_50_ values  (95% CI) |
| --- | --- | --- | --- | --- | --- | --- | --- |
|  | 0 µg/mL | 10 µg/mL | 50 µg/mL | 100 µg/mL | 200 µg/mL | 500 µg/mL |  |
| MCF7 | 100 ± 12.3 | 84.3 ± 7.5 | 59.8 ± 10.3* | 33.7 ± 3.3** | 17.4 ± 3.4** | 9.3 ± 2.5** | 60.5 ± 11.4  (49.5 - 71.9) |
| MKN45 | 100 ± 6.9 | 85.7 ± 5.0 | 77.6 ± 7.1 | 50.2 ± 9.3* | 25.5 ± 8.0** | 20.1 ± 5.8** | 105.1 ± 22.5  (85.9 – 127.6) |
| AGS | 100 ± 9.7 | 70.9 ± 7.5* | 62.2 ± 1.3* | 58.7 ± 6.8* | 28.7 ± 4.8** | 16.0 ± 5.3** | 80.8 ± 29.7  (56.8 – 110.5) |
| Note: Cell proliferation values are presented as % Mean ± SD, * p < 0.05, ** p < 0.01 vs. control. T test. | | | | | | |  |
